# The effect of uncertainty on pain decisions for self and others

**DOI:** 10.1002/ejp.1940

**Published:** 2022-04-01

**Authors:** Leyla Loued‐Khenissi, Sandra Martin‐Brevet, Luis Schumacher, Corrado Corradi‐Dell’Acqua

**Affiliations:** ^1^ Theory of Pain Laboratory Department of Psychology Faculty of Psychology and Educational Sciences (FPSE) University of Geneva Geneva Switzerland; ^2^ Swiss Center for Affective Sciences University of Geneva Geneva Switzerland; ^3^ Geneva Neuroscience Center University of Geneva Geneva Switzerland

## Abstract

**Background:**

Estimating others’ pain is a challenging inferential process, associated with a high degree of uncertainty. While much is known about uncertainty’s effect on self‐regarding actions, its impact on other‐regarding decisions for pain have yet to be characterized.

**Aim:**

The present study exploited models of probabilistic decision‐making to investigate how uncertainty influences the valuation and assessment of another’s pain.

**Materials & Methods:**

We engaged 63 dyads (43 strangers and 20 romantic couples) in a task where individual choices affected the pain delivered to either oneself (the agent) or the other member of the dyad. At each trial, agents were presented with cues predicting a given pain intensity with an associated probability of occurrence. Agents either chose a sure (mild decrease of pain) or risky (50% chance of avoiding pain altogether) management option, before bidding on their choice. A heat stimulation was then issued to the target (self or other). Decision‐makers were then asked to rate the pain administered to the target.

**Results:**

We found that the higher the expected pain, the more risk‐averse agents became, in line with findings in value‐based decision‐making. Furthermore, agents gambled less on another individual’s pain (especially strangers) and placed higher bids on pain relief than they did for themselves. Most critically, the uncertainty associated with expected pain dampened ratings made for strangers’ pain. This contrasted with the effect on an agent’s own pain, for which risk had a marginal hyperalgesic effect.

**Discussion & Conclusion:**

Overall, our results suggested that risk selectively affects decision‐making on a stranger’s suffering, both at the level of assessment and treatment selection, by (1) leading to underestimation, (2) privileging sure options and (3) altruistically allocating more money to insure the treatment’s success.

**Significance:**

Uncertainty biases decision‐making but it is unclear if it affects choice behavior on pain for others. In examining this question, we found individuals were generally risk‐seeking when faced with looming pain, but more so for self; and assigned higher monetary values and subjective ratings on another’s pain. However, uncertainty dampened agents’ assessment of a stranger’s pain, suggesting latent variables may contradict overt altruism. This bias may underlie pain underestimation in clinical settings.

## 1 INTRODUCTION

Understanding others’ pain is a challenging process. Unlike other medical conditions, pain is difficult to quantify objectively and is often diagnosed through indirect information. Consequently, caregivers base their decisions on pain under uncertainty, which can negatively impact their own (Bovier & Perneger, 2007), as well as their patients’ well‐being.

Uncertainty has been extensively studied in economic decision‐making (Johnson & Busemeyer, 2010; Loued‐Khenissi & Preuschoff, 2020; Preuschoff et al., 2013) particularly in the form of ‘risk’, the uncertainty expected from available information. Previous studies have found that people avoid risk when facing gains, whereas they seek it when facing monetary loss (Kahneman & Tversky, 1979). This risk‐aversion is less pronounced when making decisions on behalf of other people (Polman & Wu, 2020; Lu et al., 2018; Ogawa et al., 2018); but see (Loued‐Khenissi & Corradi‐Dell'Acqua, 2020). It is still unclear, however, whether risk influences pain decisions as it does in economic settings (Baliki et al., 2010).

Pain is a subjective experience that can be modulated by factors like belief, expectancy (Atlas et al., 2010; Geuter et al., 2017; Sharvit et al., 2019), and uncertainty (Ploghaus et al., 2003). Pain might be ‘inferred’ according to a predictive coding process (Büchel et al., 2014; Ongaro & Kaptchuk, 2019; Seymour, 2019; Tabor & Burr, 2019) whereby an agent integrates nociceptive inputs with pre‐existing knowledge to estimate potential body damage (Morrison et al., 2007). A prediction on an incoming stimulus can be formalized as an expected value, or average predicted outcome. This quantity is captured by weighting a predicted stimulus value (e.g. moderate pain) by its probability of occurrence. Within the predictive coding framework, studies investigating the role of uncertainty on pain experience have unveiled mixed results, with some finding hyperalgesia (Taylor et al., 2017; Yoshida et al., 2013) and others a hypoalgesic effect (Hoskin et al., 2019).

It is also unclear how uncertainty affects decisions on others’ pain (Vlaev et al., 2017). One model suggests that inferring another's state may occur by recruiting the same mechanisms underlying first‐hand experiences (Bernhardt & Singer, 2012; Furnham & Boo, 2011). This strategy appears more pronounced when the other is someone close, rather than a stranger (Hein et al., 2010; Xu et al., 2009). Hence, although never developed in the context of risky decision‐making, this account suggests that uncertainty influences decisions on others’ pain similarly to one's own. The question of how uncertainty affects decisions made on others’ pain can inform along two dimensions: ‘rationality’ and objectivity. Expected utility theory suggests that rational choice selection should maximize reward and minimize pain, although deviations are consistently found in human decision‐making. Objective decision‐making on pain should predict the same choices made for one's self and others. Deviations from both rationality and objectivity can inform on such questions as differences in pain management for different targets (such as self or another).

Here, we investigated uncertainty's role in decision‐making on pain in others by applying an expected value model of pain in a probabilistic task where individuals chose between gambles or sure options for pain management. Participants bid on selected choices, and subsequently rated the pain event's intensity. This paradigm tested the unique effect of uncertainty on participants’ decisions and subsequent assessment of another's pain. We also manipulated the other's identity, with one group acting for romantic partners, and another for strangers. Finally, we included a condition where pain was directed towards participants themselves. We expected uncertainty to (1) privilege gambles, as it does for monetary losses (Baliki et al., 2010), and (2) lead to hyperalgesia (Taylor et al., 2017; Yoshida et al., 2013); but see (Hoskin et al., 2019). We also hypothesized that uncertainty effects differ according to the target's social proximity, with agents acting for partners as they would for themselves, but differently for strangers.

## 2 METHODS

### 2.1 Participants

A total of 65 dyads were recruited for the experiment. The study was open to the general population. Invitation to participate was advertised via paper flyers and online platforms linked to the University of Geneva and the Campus Biotech, in Geneva, Switzerland. Participants were required to be in good general health; and be of age 18 and over. Exclusion criteria included excessive consumption of alcohol, use of psychotropic substances and psychiatric or neurological conditions. The study was conducted in an experimental room at the Campus Biotech in Geneva, Switzerland, between June and December 2020. As the study was conducted during the SaRS‐COV2 pandemic, participants and experimenters were required to wear masks throughout the experiment.

We recruited twenty dyads consisting of heterosexual romantic couples (>6 month long relationship), including twenty Deciding Agents (DA; 10 females, mean age = 21.18 ± 2.78 SD) and twenty romantic partners acting as Passive Targets (PT; 10 females, mean age = 20.80 ± 4.07 SD). The remaining dyads were pairs of individuals unknown to one another. These included 43 DAs (26 females, mean age = 23.78 ± 6.17 SD) that were paired with independently recruited 43 PTs (29 females, mean age = 24.42 ± 5.46 SD). All participants were given consent forms to read, understand and sign, before being assigned randomly to their role (DAs, PTs) and entering the experimental room. The study was approved by the ethical committee of the Geneva Canton and was conducted in accordance of the Declaration of Helsinki.

### 2.2 Pain stimulations

Nociceptive radiant heat stimuli were administered to the back of participants’ right hand via infrared neodymium: yttrium–aluminum–perovskite laser system (Nd: YAP; Stimul 1340; El. En, Florence; wavelength 1.34 µm; pulse duration 4 ms, beam diameter 6 mm). Laser pulses at this wavelength are expected to selectively trigger Aδ and C fiber nociceptive terminals located in the superficial layers of hairy and glabrous skin (Iannetti et al., 2006). Since these fibers have different conduction velocity, participants first experience an initial pricking pain due to Aδ‐fiber stimulation, and subsequently a C‐fiber‐related burning experience.

For each participant, we identified three levels of energy, ranging from 1.25 to 2.5 J, aimed at evoking three different levels of pain. These two points were chosen to ensure the threat of pain (such that decisions made by the participant were relevant to the pain cue) while maintaining at the same time participants’ safety.

The calibration procedure consisted of delivering a random sequence of all possible laser pulses between 1.25–2.25 J (in 0.25 step intervals) 3 times each. Participants rated the pulse received on a scale, ranging from 0 (corresponding to ‘no pain’) to 10, where 1 was explicitly cast as the lowest pain experienced. The scale displayed the anchors ‘0’ and ‘10’ as well as interval tick‐marks with no associated numbers. Although slightly different from previous studies employing similar laser stimulations (where the pain threshold is mapped to around ~4 of the 10‐point scale, [Hagiwara et al., 2020]), our approach allowed us to exploit the full scale to identify three energy levels characterized by different, and well distinguishable, pain levels. At each stimulation, participants selected the position of the scale that corresponded to their judgment, which was then automatically converted to a value ranging from 0 to 10. The average ratings (from the three repetitions) of all possible energy levels was used to identify three stimulations corresponding approximately to scale values of 3, 5 and 7 (low, medium and high pain). In cases where two subjective pain levels were associated with the same energy, 0.25 J was added to the highest subjective pain level. Each calibration procedure began with the highest possible stimulation (2.5 J), to acclimate the participant to the sensation of the laser stimulation and offer them the opportunity to retract their consent; the rating for this initial stimulation was discarded from subsequent ratings. The code for the calibration procedure can be found in the following repository: https://github.com/LLouedKhen/PainCalibrationLaserStimulHand.

Following the calibration procedure, each pain level (low, medium and high) pain was presented to the participant a final time. During laser administration procedures, all occupants in the room were required to wear laser safety goggles. Calibrations were performed sequentially, first with the PT, followed by the DA. During calibration, the other member of the dyad was seated in a curtained off area of the room.

### 2.3 Task procedure

**FIGURE 1:**
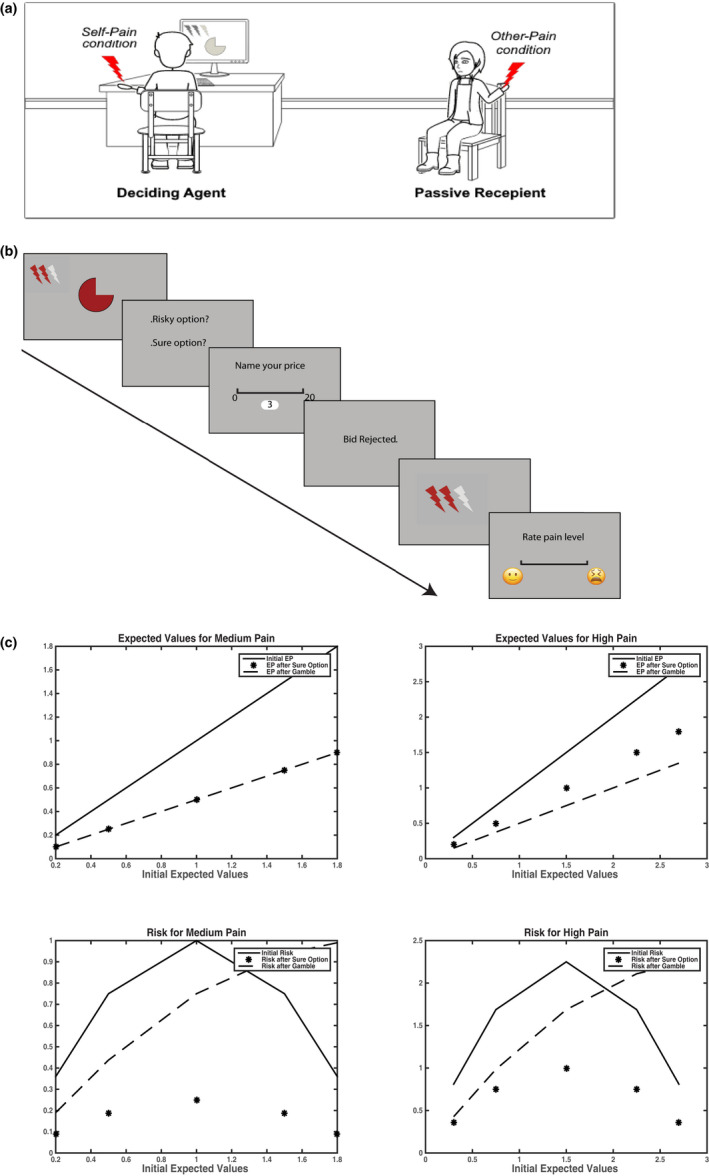
Task set‐up, trial structure and decision variables. (a) A schematic representation of the experimental setup. DAs were seated in front of a screen where the trials were displayed. They received nociception stimulation during their block. PTs were seated nearby and received nociception stimulation in the alternative block. Block order for targets was randomly assigned across subjects. (b) The trial structure is shown here. DAs are first presented with a probabilistic pain cue, where pain intensities are denoted by either 2 or 3 red lightning rods indicating medium or high pain and a pie chart representing the probability of pain occurrence in red. They are subsequently asked to select either a risky option (50% chance of avoiding pain altogether) or a sure option (pain will be surely lessened by 1 level). Then they are asked to bid on their choice, using their endowment, on a scale of 0 to 20 MUs (0–2 CHF). If they bid equal or higher than a hidden, random reserve price, their choice is selected and applied to the initial expected pain. If their bid is rejected, their expected pain does not change from the initial value. Pain is then delivered according to the resultant probabilities and intensities, either to the self or the other. Finally, DAs were asked to rate the received pain on a visual analogue scale. (c) Expected values and risk for gambles and sure options. For medium pain intensities, both options yield equivalent expected values following choice, while gambles are more advantageous for high pain. Risk values following sure choices are always lower than initial risk; this decrease in risk does not occur systematically for gambles

Following the calibration procedure, participants were both seated in the experimental area of the room and told to refrain from communicating with one another (Figure 1a). The DA was seated in front of the experimental monitor, with the experimenter and laser machine to her right; and the PT to her left. Participants performed three practice trials of the experiment, after which they were asked if they had any questions. Following the practice period, participants played 2 blocks of 10 trials of the task, with either self or other designated as the target of the painful events.

The task included 2 blocks of 10 trials each of a probabilistic pain task. Participants were endowed with 220 monetary units (MU), corresponding to 22 CHF, at each block. At each trial, participants were presented with an image of a probability, represented as a red pie chart on a gray background, together with an icon of 2 or 3 lightning bolts, representing a stimulus of medium or high pain respectively (Figure 1b). The probabilities were set to 0.1, 0.25, 0.5, 0.75 or 0.9. This image was displayed for 5 s. Following this, participants were asked whether they wanted to choose a gamble, with a 50% chance of avoiding pain altogether, or whether they preferred a sure option, with a sure reduction of pain intensity by one level (high to medium, or medium to low). Although our hypothesis could in principle be tested also by using other choice parameters, we settled on 50% (for gambles) and one pain level reduction (for sure choices) to facilitate participants’ understanding of the choices offered and to avoid additional levels of complexity in the task. Participants were given 5 s to respond using a button response box. Trials with no responses incurred a penalty of 2 MUs. Following their choice selection, participants were asked to bid on their selected option, by entering a price ranging from 0 to 20 MUs (see Figure 1b). Participants had 7 s to provide a response, which was validated by pressing the enter key. Participant bids were then compared to a randomly generated reserve price between 0 and 20 MUs and were either accepted, with the bid deducted from their endowment, or rejected. Participants were informed of the outcome of their bidding through a dedicated screen message that was displayed for 2 s. The trial outcome was then delivered. When bids were rejected, trial outcomes resulted from the intensity and probability displayed at the beginning of the trial. Accepted bids on the other hand allowed for the purchase of the choice made, either reducing pain probability (for gambles) or intensity (for sure options). The laser stimulation lasted 4 ms and was presented simultaneously with an icon of either red lightning bolts (if the initial pain intensity was delivered); orange lightning bolts (if pain was reduced) or grey lightning bolts (for no pain). The icon was presented for 6 s. Finally, participants were asked to rate the pain of the trial outcome on a 10‐point scale. The scale and the response delivery were similar to the one used in the calibration, with the exception that the anchors used here consisted of emojis (happy for no pain, and unhappy for most pain) with intermediate tick‐marks in between. Participants had an unlimited time to assess the pain. Trials were bookended with a jittered 2–5 s inter‐trial interval.

PTs remained passive during the whole experiment, without interacting with DAs and/or the experimenters. They were the targets of the painful stimulation in one of the two experimental blocks and were instructed to refrain from explicitly communicating, verbally or otherwise, with the DAs during the experiment (PTs were also told not to force stoicism during the pain stimulation). DAs and PTs were paid a fixed amount of money for their time (30 CHF). In addition, DAs were paid a sum corresponding to the payout of one randomly selected experimental block. The whole experimental session lasted approximately 1 h.

### 2.4 Modeling expected pain and uncertainty

Our task was based on pre‐existing models testing the effect of uncertainty on economic decision‐making, which was adapted to the domain of pain assessment and treatment. Within this framework, we expected participants to estimate the expected pain by weighting the intensity with its probability of occurrence. This allowed for the mathematical definition of as the variance of the expected pain (Markowitz, 1952). These formalizations offer the added benefit of increased precision in assessing the effects of uncertainty on participants’ responses beyond clear‐cut categorizations such as probabilistic (vs. certain) or expected (vs. unexpected) stimuli. Moreover, changes in either intensity or probability can be quantified on the same expected value function at the point that either gambles or sure choices might (despite their inherent difference in terms of uncertainty) lead to comparable expected outcomes (see Figure 1c for an example).

Expected pain and pain risk were formalized by the mean‐variance theorem. Within this framework, initial expected pain (*EP_0_*) was defined as follows

$$EP_{0}=p_{0}*P$$

where *P* refers to the stimulation *intensity* (ranging from 2 to 3, corresponding to medium and high stimulations respectively) and *p_0_* to the associated *probability* (ranging from 0.1 to 0.9). According to this formula, *EP_0_* reaches its’ peak when both *P* and *p_0_* are the highest, as participants expect a stimulation which is both highly intense and highly probable. Risk was taken as the variance of the expected values of the two possible outcomes:

$$Risk_{0}=p_{o}*{\left(p_{o}*P\right)}^{2}‐{\left(p_{o}*P\right)}^{2}$$

*Risk_0_* reaches its’ peak when *P* is highest and *p_0_* = 0.5, as participants are confronted with two opposite outcomes (high pain vs. no pain) which are equally likely. *EP_0_* and *Risk_0_* represent expected pain and risk estimates at the beginning of the trial. When bids were accepted, these values were updated as follows:

$$EP_{1}=\left(\frac{p_{o}}{2}\right)*P\;\left(\text{gamble}\right)$$

$$EP_{1}=p_{0}*\left(P‐1\right)\;\left(\text{sure option}\right)$$

$$Risk_{1}=\left(\frac{p_{o}}{2}\right)*{\left(\left(\frac{p_{o}}{2}\right)*P\right)}^{2}‐{\left(\left(\frac{p_{o}}{2}\right)*P\right)}^{2}\;\left(\text{gamble}\right)$$

$$Risk_{1}=p_{o}*{\left(p_{o}*\left(P‐1\right)\right)}^{2}‐{\left(p_{o}*\left(P‐1\right)\right)}^{2}\;\left(\text{sure option}\right)$$

As seen in the formulas above, gambles affect only *p_0_*, decreasing the likelihood of pain. On the other hand, sure options only affect *P*, in which case participants are sure that any stimulation received will be milder than the initial one. Importantly, when bids are accepted, both gambles and sure options systematically reduce expected pain (especially for gambles on high pain). This is not the case of risk which decreases systematically only when a sure option is accepted (Figure 1c). Instead, when bids were refused, expected pain and risk remain unchanged (*EP_1_ *=* EP_0_* and *Risk_1_ *=* Risk_0_*).

### 2.5 Data analysis

In a first instance, we analyzed mean payout (how much money participants retained); mean willingness to pay (*WTP*, the bid made for each trial); and mean pain outcome (the actual stimulation delivered, using a scalar ranging between 0 [no pain], 1 [low], 2 [medium] and 3 [high]). We also derived a measure of risk aversion, over each individual's utility curve (Arrow, 1965; Pratt, 1964). These represent global measures reflecting participants’ overall decision strategy for self and others.

Subsequently, we modeled single‐trial decisions as a function of expected values of pain and pain risk, following the fomulae reported above. To determine how expected pain and risk modulate decision‐making on sensitivity to pain, across self and others, we fit three models testing participants’ choice, bids and pain assessment respectively. In Model 1, we examined participants’ choices, coded as a dichotomous variable (0 = sure option, 1 = gamble), fit against *EP_0_* and *Risk_0_* as continuous predictors, *Target* (Self, Beloved and Stranger) as fixed factor, and the interaction terms Target*EP_0_ and Target*Risk_0_. In Model 2, we examined willingness to pay (*WTP)* for the previously‐selected choice fit against *EP_0_*, *Risk_0_*, *Target*, *Choice* (0 = sure option, 1 = gamble), as well as Target*EP_0_, Target*Risk_0_ and Target*Choice. In Model 3, we analyzed pain ratings as a function of *Pain Outcome*, *Risk_1_*, *Target*, *WTP*, as well as Target*Pain Outcome, Target*Risk_1_ and Target*WTP. The analyses were performed using a mixed model scheme, with subjects’ identity specified as a random factor. For Model 1 we performed a generalized linear mixed model with binomial distribution and Laplace approximation, whereas for Models 2–3, we performed a linear mixed model. In all models (Table 1), DA’s sex, as well as sex heterogeneity across the dyad, was included as nuisance variables.

**TABLE 1:** Description of three models used to examine choice behavior at different time points of the decision process. EP: expected pain; DA: deciding agent; WTP: willingness‐to‐pay; PO: pain outcome.

Model	Outcome variable	Main predictors	Nuisance variables	Random effect
1	*Choice* (0= Sure 1 = Gamble)	*EP_0_* *Risk_0_* *Target* Target*EP_0_ Target*Risk_0_	Sex DA Same sex dyad	Subject
2	*Willingness*‐*to*‐*pay* (WTP)	*EP_0_* *Risk_0_* *Choice* *Target* Target*EP_0_ Target*Risk_0_ Target*Choice	Sex DA Same sex dyad	Subject
3	*Pain rating*	*Pain outcome [PO]* *Risk_1_* *WTP* *Target* Target*PO Target*Risk_1_ Target*WTP	Sex DA Same sex dyad	Subject

All experimental code was written in Python 3.7 using PsychoPy (Peirce et al., 2019), and analyses were conducted in Matlab 2017 and Python 3.7.

## 3 RESULTS

**FIGURE 2:**
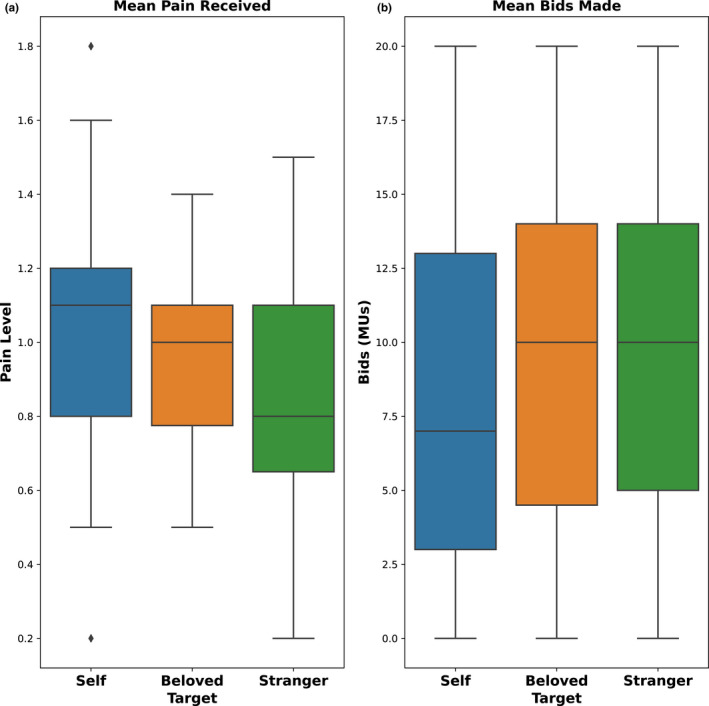
Mean pain intensity delivered and mean bid made as a function of decision target. Results show that pain delivered decreases with increasing bids, underscoring the link between pain and monetary valuation

A total of 65 dyads participated in the study. One dyad was excluded due to hardware malfunction and another due to the participant's difficulty in understanding the task. The overall sample included in the analysis was comprised of 63 dyads. Among the selected 63 dyads (20 romantic couples and 43 pairs of unknown individuals), DAs received an average payout of 16.10 CHF (sd = 4.57). The average pain delivered was 1.04 (sd = 1.20), 0.94 (sd = 1.20) and 0.88 (sd = 1.15) for Self, Beloved and Stranger, respectively. Average bids across targets were 8.06 (sd = 5.99), 9.30 (sd = 5.98) and 9.53 (sd = 5.63) MUs for Self, Beloved and Stranger. Participants bid less on themselves (*F*(2,1256) = 9.06, *p* < 0.001) and undergo marginally more pain than others (*F*(2,1256) = 2.45, *p* = 0.086; Figure 2).

**FIGURE 3:**
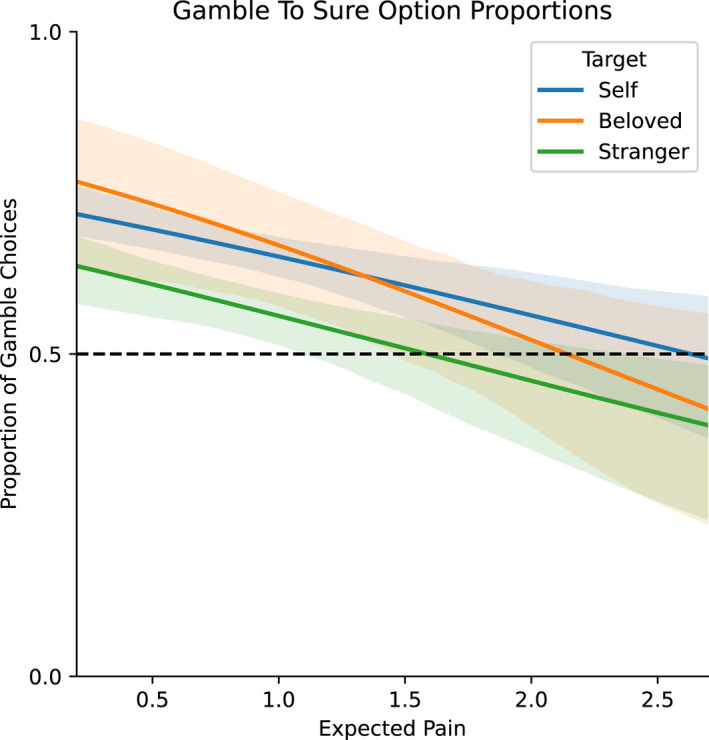
Proportion of gambles to sure options plotted as a function of expected pain values for each pain target. These values were fitted by a linear function, for each decision target. All targets prompt more gambles with lower values of expected pain, a preference that shows a linear decrease as expected pain values increase

Choices on pain followed expected value and prospect theory, with participants selecting risky options with low values of expected pain and increasing the selection of sure options linearly with higher levels of expected pain (Figure 3). Linear fitting of the grand mean of gamble to sure option proportions against expected valued of pain yield the following correlation coefficients: *r* = 0.63 for self; *r* = 0.50 for the Beloved; and *r* = 0.62 for the Stranger. Participants selected risky options more often across all trials and targets (59.17%), a tendency reflected in overall risk aversion values.

### 3.1 Model 1

We modeled choice (gambles vs. sure options) with a binomial regression, and found a main effect of *EP_0_* (*t* = 3.96, *p* < 0.001) and a main effect of target Stranger (*t* = 2.56, *p* = 0.011) both of which predicted a sure option selection (Figure 3).

### 3.2 Model 2

**FIGURE 4:**
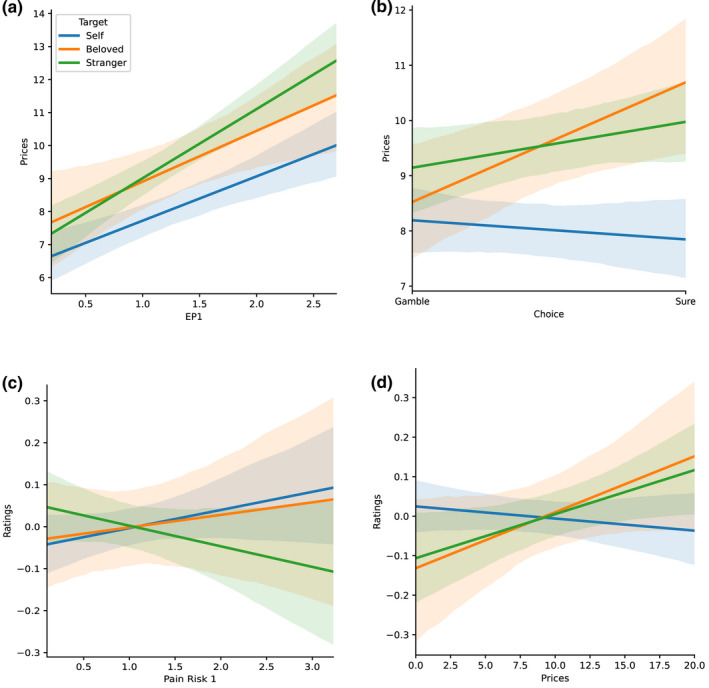
Linear regression plots for pain valuation and assessment. a) Bids plotted as a function of expected pain, for each decision target. All targets incur higher bids with higher expected pain. b) Bids plotted as a function of choice selection. Here, it is seen that sure options are valued more than gambles but only for others. c) Pain ratings plotted as a function of pain risk. Risk dampens pain ratings for strangers, while slightly inflating that of self and beloved. d) Pain ratings plotted as a function of bids. Here, we see an inflation of pain ratings as a function of previously made bids, but for others only

We then examined WTP and found a positive main effect of *EP_0_* (*z* = 7.15, *p* < 0.001) as well as a main effect of *Target*, whereby individuals bid more for others (both Beloveds, *z* = 3.13, *p* = 0.002 and unknown others, *z* = 6.11, *p *< 0.001) than for themselves (Figure 4a). A Target*Choice interaction was also found, with higher bids made on sure options for the Beloved (*z* = 2.49, *p* 0.013) and the Stranger (*z* = 2.09, *p* = 0.037; Figure 4b). Finally, WTP was significantly lower in males, relative to females (*z* = −2.86, *p* = 0.004). No other effect was found to be significant.

### 3.3 Model 3

Finally, we modeled individual pain ratings, for which we found a significant main effect of *Pain Outcome* (*z* = 23.16, *p* < 0.001). We also found a main effect of *Target*, with higher ratings made for the Beloved (*z* = 3.46, *p* = 0.001) and the stranger (*z* = 7.87, *p* < 0.001) than for oneself. We also found a significant Target*Pain Outcome interaction, suggesting a more pronounced effect of the stimulation on the Beloved (*z* = 2.64, *p* = 0.008) and the Stranger (*z* = 7.54, *p* < 0.001) compared with oneself. Importantly, whereas participants based their own pain assessment also on the nociceptive stimulation on their skin, others’ pain was inferable only from visual cues (lightning rods) displayed on the screen, and therefore might be less susceptible to habituation/desensitization.

In addition to the above, we found a marginal main effect of *Risk_1_*, suggesting pain risk enhances one's own pain perception (*z* = 1.77, *p* = 0.076). We also found a significant Target* Risk_1_ interaction, revealing that pain risk dampens ratings on a stranger's pain (*z* = −2.58, *p* = 0.010). Importantly, this effect was not found for the Beloved, whose ratings were impacted by *Risk_1_* to a similar degree as they are for oneself (*z* = −0.445, *p* = 0.657; see Figure 4c). Finally, we found a significant Target* WTP interaction, suggesting that, relative to the self, the ratings of the Beloved's (*z* = 6.437, *p *< 0.001) and stranger's pain (*z* = 5.84, *p* < 0.001) were stronger in trials in which more money was allocated (Figure 4d). No other effect was found to be significant.

## 4 DISCUSSION

In this study, we investigated the role of risk in decisions made on another's pain. By exploiting models of probabilistic decision‐making, we identified differences and commonalities between self‐ and other‐regarding risky choices on pain. First, we found that participants were generally risk‐seeking overall. However, participants chose sure options more frequently with increasing values of expected pain. Individuals also selected sure options more frequently when facing a stranger (as opposed to the self or a partner), and assigned a higher monetary value to other‐related choices, especially sure options. Finally, risk downgraded the assessment of a stranger's pain, in striking contrast with self‐related pain, where we found a marginal hyperalgesic effect (Yoshida et al., 2013) but see (Hoskin et al., 2019). Overall, models for risk‐based decision‐making proved useful in explaining decision‐making on pain.

### 4.1 Decisions for others’ pain are less risky

Decisions in our study were risk‐seeking, an effect which is in line with studies on different kinds of negative rewards, ranging from monetary loss (Kahneman & Tversky, 1979) to disease prevention (Attema et al., 2016; Loued‐Khenissi & Corradi‐Dell'Acqua, 2020). In all these cases, participants became more risk‐averse as the expected value of the negative event increased, an effect also confirmed in our study (Figure 3). Risky choices, however, were significantly tempered when acting for a stranger (Figure 3). This contradicts studies on economic decisions documenting slightly more risk‐seeking behavior for others than for oneself (Leonhardt et al., 2011; Polman & Wu, 2020), but see (Vlaev et al., 2017). Individual choices may be influenced by the inter‐personal relationship between the agent and the target, such as subjective responsibility or associated negative response (Lu et al., 2018; Pahlke et al., 2015). From this perspective, our results highlight the nature of the relationship between self and other, with participants exhibiting more risk aversion when deciding for strangers, but no differences when acting for romantic partners.

Consistent with risk preference findings above, participants allocated more money for sure options, higher expected pain and decisions for others (Figure 4a). However, in contrast to choice selection, both romantic partners and strangers prompted a higher willingness to pay for pain avoidance, suggesting the disutility of another's pain is greater than one's own. Critically, when explicitly modeling the effect of the previous choice on willingness‐to‐pay, we found that sure options were valued more than gambles, but only for others (partners and strangers alike; Figure 4b). Hence, participants’ behavior towards others’ pain is not a sign of disengagement, but rather a willingness to prevent suffering at a personal cost. Previous studies have uncovered similar other‐regarding costly choice behavior in relation to pain (Crockett et al., 2014; Hein et al., 2011), as well as a positive influence of uncertainty for others’ wellbeing in individual pro‐sociality (Kappes et al., 2018). Our data support, and extend these previous findings, by revealing that costly prosocial behavior encompasses different kinds of targets (partner vs. stranger) and exceeds the price participants are willing to pay for their own pain management.

### 4.2 Risk downgrades a stranger's pain

We also analyzed whether risk affected pain ratings. Seminal models argue that self‐pain experience is best describable in a Bayesian framework, where the brain estimates the probability of body damage, based on the integration of sensory inputs and prior knowledge (Büchel et al., 2014; Ongaro & Kaptchuk, 2019; Seymour, 2019; Tabor & Burr, 2019). Within this framework, priors with high uncertainty are expected to exert a lower influence on the posterior estimate, which in turn should be based more strongly on bottom‐up information (Hoskin et al., 2019). However, this is not what is observed in the present research, which instead revealed that risk led to a marginal hyperalgesia on one's own pain perception (as previously observed (Taylor et al., 2017; Yoshida et al., 2013)) but also to an opposite hypoalgesic effect on the pain of strangers.

One possible explanation might be that uncertainty is negatively valenced in itself, and often associated with fear and anxiety (see Yoshida et al., 2013, for a similar argument). Hence, its role in self‐pain would be comparable with that observed in the literature of emotion induction, with positive events leading to hypoalgesia and negative events to hyperalgesia (Berna et al., 2010; Loggia et al., 2008; Qiao‐Tasserit et al., 2018; Roy et al., 2008; Villemure et al., 2003; Weisenberg et al., 1998). Critically, emotion induction appears to operate on the sensitivity of another's pain in an opposite fashion than to one's own (Qiao‐Tasserit et al., 2018). This has been interpreted in light of *broaden*‐*and*‐*build* account (Fredrickson, 2004; Qiao‐Tasserit et al., 2018), according to which negative emotional states narrow one's mindset, inhibiting resources, and promoting self‐referential thought at the expense of one's social proficiency.

Our results confirm a major role of uncertainty in the assessment of pain, with a strong self‐other discrepancy observed when confronted with a stranger, but not with a romantic partner. This effect is reminiscent of that observed in the literature on pain empathy, whereby individuals are assumed to treat the suffering of others like one's own (Bernhardt & Singer, 2012; Gallese, 2003; Goldman & Vignemont, 2009), but to a lesser extent when the observed person is deemed distant from the self (Cheng et al., 2010; Hein et al., 2010; Xu et al., 2009). Previous studies have already shown how brain models of pain empathy can explain decisions made on behalf of others, such as altruistic monetary allocations (Tomova & Majdand_ic et al., 2017) or clinical pain diagnosis (Corradi‐Dell'Acqua et al., 2019). Thus, pain‐related decision‐making might be partly influenced by the agents’ empathetic response to the suffering of close others, which makes them susceptible to risk in a similar way to self‐related events.

### 4.3 Implications for pain management practice

Our results shed light on uncertainty's role in pain assessment and management, with relevant translational implications for healthcare practice. In particular, the ‘stranger’ condition in our study provides a reasonable estimate on how healthcare providers might assess and treat the pain of the majority of patients under their care. On the one hand, pain‐diagnostic cues can be characterized by a high level of uncertainty, for instance when different behavioral signs (e.g. facial expressions, posture) are inconsistent with one another, or with information from the clinical chart (presence of injury). Our study suggests that such uncertainty would promote pain underestimation, something that has been systematically observed in clinicians (Ruben et al., 2015; Rubin et al., 2015), as early as during university training (Corradi‐Dell'Acqua et al., 2021; Dirupo et al., 2021), and that was associated with considerations on the reliability of patient self‐reports (Dirupo et al., 2021; Vuille et al., 2018). On the other hand, our study also informs on the role played by uncertainty during pain management, suggesting that providers privilege mild but sure pain reductions, rather than risky but potentially more effective ones. This aversion may persist in the real world, as strong painkillers have an inherent risk for patients’ health (Butler et al., 2016; Makris et al., 2014). Concerns for analgesic side‐effects (e.g. opiophobia) is known to contribute to inadequate pain treatment in medical settings (Bennett & Carr, 2002; Bertrand et al., 2021; Corradi‐Dell'Acqua et al., 2019), as healthcare providers sometimes prioritize less effective treatments (e.g. a mild sedative) to avoid contraindications. Here we show how models derived from risk‐based decision‐making can predict this behavior for strangers, while a riskier attitude is accepted for oneself and loved ones. Further research is needed to determine if this effect of uncertainty on pain decision‐making replicates in clinicians.

### 4.4 Limitations of the study and conclusions

The scale used to assess pain intensity differed slightly from established specifications in the literature (Epker, 2013). First, participants were instructed to map first pain experiences at ~1 on a 0–10 scale, whereas previous studies do so at ~4 (e.g. Hagiwara et al., 2020). Furthermore, in the main experiment we employed a scale anchored by emojis (see also Dirupo et al., 2021). These changes were implemented to provide more accessible anchors and minimize confusion between different parts of the trial structure (Figure 1b). In addition, pain ratings were higher for others (both spouses and strangers) than for the self. This could reflect once more agents’ prosocial and altruistic concerns. Alternatively, it is possible that nociceptive stimulations might have become susceptible to habituation effects which, however, should not influence the assessment of others, as they are only based on visual information (Furnham & Boo, 2011). Furthermore, by borrowing the methodology from economic decision‐making, we presented pain diagnostic information as abstract probabilistic cues. Although advantageous for manipulating risk independently from expected pain, this approach has an ecological limitation, as such stimuli are not part of everyday clinical experience. One key difference lies in the sequence of decisions: in our paradigm decisions preceded the noxious event, whereas healthcare providers usually assess patients’ pain before selecting the appropriate treatments. Future research will need to bridge this gap, by investigating whether the same results are obtained in an experimental setting similar to that faced by physicians and nurses.

Notwithstanding, our study extends previous investigations on the role of uncertainty in pain management and assessment in several ways. First, it shows that decisions about pain mirror those observed in the literature on negative economic reward, with individuals being risk‐seeking overall but displaying a progressive attenuation of these tendencies with increasing expected pain. Second, decisions made for strangers are less risky than those for oneself and the spouse. Third, although risk leads to marginal hyperalgesia when pain is directed to one's own body, it has an opposite effect on the assessment of strangers’ pain. Overall, we found that uncertainty selectively affects decision‐making on pain, dissociating between choices made for oneself (and a loved one) from those made for a stranger.

## CONFLICT OF INTEREST

The authors declare no conflict of interest.
